# An Intelligent Computing Method for Contact Plan Design in the Multi-Layer Spatial Node-Based Internet of Things

**DOI:** 10.3390/s18092852

**Published:** 2018-08-29

**Authors:** Cui-Qin Dai, Qingyang Song, Lei Guo

**Affiliations:** 1 School of Computer Science and Engineering, Northeastern University, Shenyang 110819, China; songqingyang@mail.neu.edu.cn (Q.S.); guolei@mail.neu.edu.cn (L.G.); 2 Chongqing Key Lab of Mobile Communication Technology, Chongqing University of Posts and Telecommunications, Chongqing 400065, China; 3 School of Communication and Information Engineering, Chongqing University of Posts and Telecommunications, Chongqing 400065, China; songqy@cqupt.edu.cn (Q.S.); guolei@cqupt.edu.cn (L.G.)

**Keywords:** Internet of Things, computational intelligence, contact plan design, multidirectional particle swarm optimization, delivery time

## Abstract

Computational Intelligence (CI) has been addressed as a great challenge in recent years, particularly the aspects of routing, task scheduling, and other high-complexity issues. Especially for the Contact Plan Design (CPD) that schedules contacts in dynamic and resource-constrained networks, a suitable CI algorithm can be exchanged for a high-quality Contact Plan (CP) with the appropriate computational overhead. Previous works on CPD mainly focused on the optimization of satellite network connectivity, but most of them ignored network topology characteristics. In this paper, we study the CPD issue in the spatial node based Internet of Things (IoT), which enables the spatial nodes to deliver data cooperatively via intelligent networking. Specifically, we first introduce a Multi-Layer Space Communication Network (MLSCN) model consisting of satellites, High Altitude Platforms (HAPs), Unmanned Aerial Vehicles (UAVs), and ground stations, on which a Time-Evolving Graph (TEG) is used to illustrate the CPD process. Then, according to the characteristics of each layer in the MLSCN, we design the corresponding CPs for the inter-layer contacts and intra-layer contacts. After that, a CI algorithm named as Multidirectional Particle Swarm Optimization (MPSO) is proposed for inter-layer CPD, which utilizes a grid-based initialization strategy to expand the diversity of individuals, in which a quaternary search method and quaternary optimization are introduced to improve efficiency of particle swarms in iterations and to ensure the continuous search ability, respectively. Furthermore, an optimized scheme is implemented for the intra-layer CPD to reduce congestion and improve transmission efficiency. Simulation results show that the proposed CPD scheme can realize massive data transmission with high efficiency in the multi-layer spatial node-based IoT.

## 1. Introduction

As an important part of current and next generation networks, Internet of Things (IoT) is composed of physical devices, vehicles, home appliances, and other items embedded with electronics, software, sensors, actuators, and seamless connectivity, which enables these things to connect and exchange data [1,2,3,4,5,6,7,8,9]. In particular, IoT is deployed with satellite channels in geographically remote locations for agriculture, mining and transportation [10], and optimized data exchange in heterogeneous IoT architectures allows increasing data transmission efficiency and extending the application areas for the IoT technologies. Therefore, bringing wide-area connectivity to the IoT using spatial nodes and satellite technology is becoming an attractive solution to complementing terrestrial networks [11,12,13,14].

To provide a robust solution for the NP-hard problem, Computational Intelligence (CI) solves many optimization problems in real-world through biologically-and-linguistically-inspired techniques [15,16,17]. However, for different scenarios, the CI algorithm needs to be modified to be applicable [18,19,20]. Particularly, for data transmission in the spatial node-based IoT with limited resource, it is likely to affect the system performance seriously if the classical CI algorithm is applied bluntly [21,22]. In order to cope with intermittent connectivity caused by the orbital motion of spatial nodes, the spatial node-based IoT networks are often modeled through scheduling contacts as a temporal topology within a certain duration [23].

For the spatial nodes with limited on-board resources, the energy budget and the number of transponders make the node unable to fully utilize all the potential contacts, and there will be a conflict in contact establishment between the nodes. Contact Plan Design (CPD), which was proposed to resolve conflicts for the contact scheduling in the spatial resource-constrained IoT networks, is attracting more and more attention [24,25]. Early works mainly focused on the reliability of connections between nodes, while ignoring the time-varying nature of the topology caused by node movement [26,27]. Considering the dynamic characteristic of network topology, the space-time graph was proposed to discretize the time-varying network topology, and a sparser topology was generated from the original topology so as to reduce the total network overhead in [28,29]. To minimize the network overhead when contacts are unreliable, different reliabilities were allocated to each contact and several heuristic methods were proposed in [30]. Taking account of the limited-resources on satellites, authors in [31] proposed Fair Contact Plan (FCP) to enable fair scheduling of contacts based on the historical establishing times of each contact. To improve the transfer efficiency with low computational complexity, metaheuristics were introduced to CPD [32,33]. A Time-Evolving Graph (TEG) was introduced to characterize the data acquisition and delivery process while maximizing delay-constrained throughput in [34]. In view of the dynamic change of contact capacity, authors in [35] treated CPD as a queue-stability related stochastic optimization problem, and designed the Contact Plan (CP) in a slot-by-slot manner. Unfortunately, almost all of aforementioned CPD schemes were proposed for the resource-constrained satellite networks, which could hardly meet the requirements of massive and timely data transmission.

The intelligent networking with different space platforms, which aims to achieve continuous and low-delay transmission of massive data, is becoming an attractive and hot research field. In the scenario of intelligent networking, more low-altitude nodes such as Unmanned Aerial Vehicles (UAVs) and High Altitude Platforms (HAPs) are introduced to perform collaborative communications with traditional satellite nodes [36,37]. Through the cooperation between spatial nodes in different layers, a Multi-Layer Space Communication Network (MLSCN) can massively transfer data with low latency. Especially in a MLSCN consisting of UAVs and HAPs, flexible UAVs can cope with the burst of massive data transmission when hot events happen, and a stable network can be built by almost stationary HAPs to ensure the reliability of data transmission. However, the communication environment between nodes varies from layer to layer, and the motions of nodes are also taken into account when establishing the inter-layer links, which further increase the difficulty of CPD.

In this paper, we study the CPD in a MLSCN by introducing an intelligent computing method. We divide the CPD into intra-layer CPD and inter-layer CPD, and schedule the CPs in the delivery order of data. Specifically, a MLSCN model containing ground stations, UAVs, HAPs, and satellites, is presented first, and TEG is introduced to illustrate the difference between contact topology and CPs in MLSCN. Then, different CPD schemes are proposed for different inter-layer and intra-layer, respectively. For inter-layer contacts, a CI method named Multidirectional Particle Swarm Optimization (MPSO) is proposed to schedule conflicting contacts in an iterative manner. To ensure that the particles are able to search the solution domain as completely as possible during the initial stage of iterations, the gird-based initialization in MPSO maximizes the diversity of the CPs in the initial solution set. In addition, to avoid the particles’ falling into local optimization trap, the quaternary optimization of MPSO can adjust the direction of searching particles based on the optimal position and the worst position.

For intra-layer CPs, since the data in UAVs is transferred cooperatively through HAPs and satellites, a load balancing strategy is introduced in the UAV layer to deliver the data to the higher layers as soon as possible. The main concern of the HAP layer and the satellite layer is to relay the data from the lower layer and transfer it to ground stations. Therefore, we adopt the modified Dijkstra algorithm and Dynamic Virtual Topology Routing (DVTR) algorithm in the CPD of the HAP layer and the satellite layer. The simulation results show that our proposed CPD scheme can deal with the dynamic topology, and realize real-time, high-efficiency, and massive data transmission.

The remainder of this paper is organized as follows. In Section 2, a MLSCN consisting of ground stations, UAVs, HAPs, and satellites is introduced, the data transfer process is characterized in a TEG manner, and a problem description is presented. Then, MPSO is proposed for the inter-layer CPD in Section 3, and optimized strategies for the intra-layer CPD of each network layer are introduced in Section 4. Finally, simulation results are presented and analyzed in Section 5 and conclusions are drawn in Section 6.

## 2. System Model and Problem Description

### 2.1. Network Topology

In our envisioned scenario, the MLSCN contains four kinds of nodes, which are satellites, HAPs, UAVs, and ground stations. Space nodes of the same type can build intra-layer networks, which means that the MLSCN has a total of three intra-layer networks. The space nodes of adjacent layers can be connected through inter-layer links; UAVs are connected to HAPs through Platform-Vehicle Links (PVLs), and HAPs are connected to satellites through Satellite-Platform Links (SPLs). When the destination nodes, i.e., the ground stations, are within the visual range of the space nodes, the MLSCN can build links to the ground, which are Vehicle-Ground Links (VGLs), Platform-Ground Links (PGLs), and Satellite-Ground Links (SGLs), respectively.

**Figure 1:**
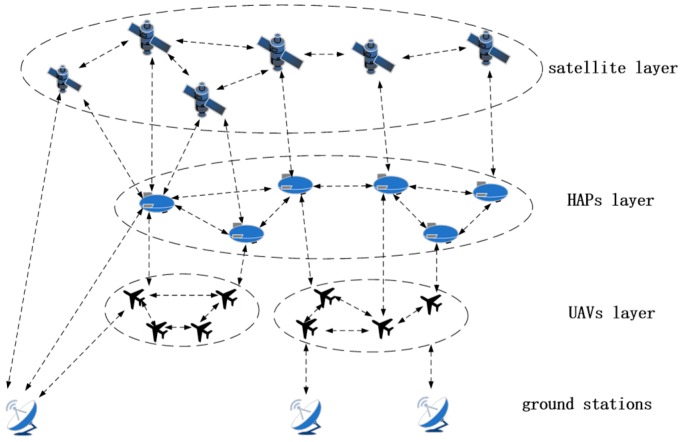
Multi-Layer Space Communication Network.

Taking into account the complexity of data acquisition in the hot-event case, UAVs are the only data source nodes in the MLSCN in Figure 1. A UAV cluster consists of multiple UAVs, and UAVs in the same cluster can connect to each other. Although the flexibility of UAVs ensures that it can perform high-intensity data collection for specific areas, the limited range makes it difficult to efficiently deliver data to the destination nodes. At this point, the HAP network can act as a data mule to assist UAVs to transfer data more efficiently. With a wider coverage, satellites can assist MLSCN in reducing the number of hops and improving the transmission efficiency.

### 2.2. Time-Evolving Graph with Limited Capacity

Since all inter-layer links except PGLs show the characteristic of intermittent connection, the network topology is time-varying. In view of this, a time-evolving graph is introduced to characterize the time-evolving nature of the network and illustrate an example of CP.

**Figure 2:**
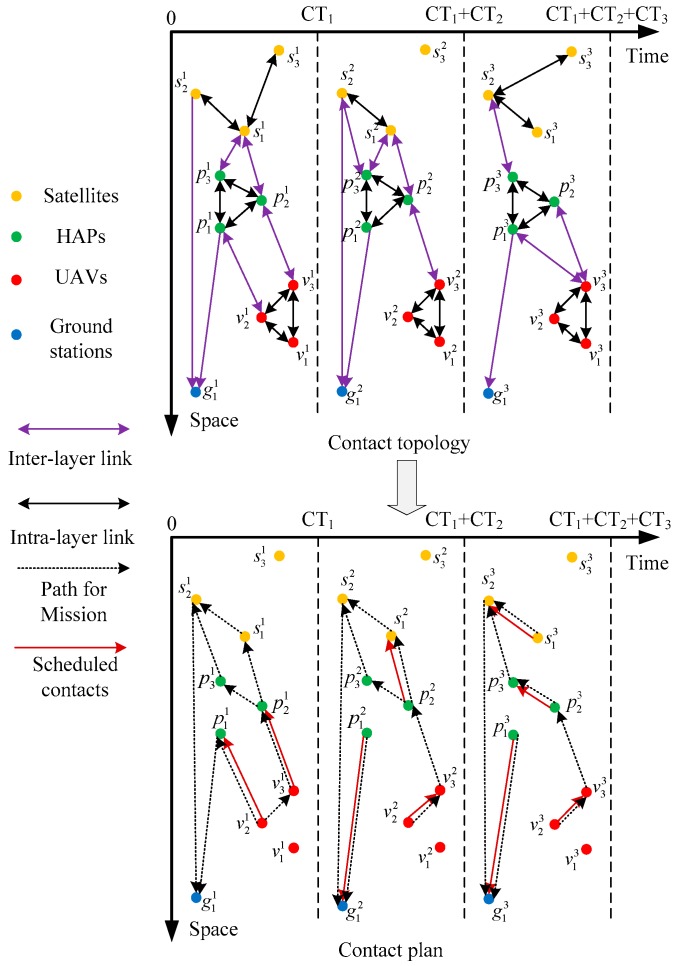
A contact topology and a contact plan in TEG manner.

As shown in Figure 2, the network topology over time is characterized by TEG. Where $g_{n},v_{n},p_{n},s_{n}$ represent Earth stations, UAVs, HAPs, and satellites, respectively. The subscript of the node indicates the node identifier, and the superscript is the state number of the node. For example, $s_{2}^{1}$ indicates the satellite 2 at the state 1. $C_{1},C_{2},\cdots ,C_{n}$ represent the state of the network, $CT_{1},CT_{2},\cdots ,CT_{n}$ are the corresponding contact time of each state.

In the contact topology of Figure 2, the change of network topology in TEG is clarified from two perspectives. In the perspective of time, the network topology updates when the connection status between nodes changes. In the other perspective of space, the intra-layer links of HAPs are stable due to the stable position while the intra-layer links of the satellite layer are intermittent connected due to the movement of satellites. Considering the limitation of the number of transponders and the energy budget, only one contact can be established by spatial nodes at the same time, which is shown in Equation (1):

(1) $$\sum_{n=1}^{N_{s}+N_{h}+N_{v}}Y_{c,n,m}=\{0,1\}\;\;\forall c,\;m=s,h,v$$

where s,h,v indicate the satellites, HAPs, and UAVs respectively, and $N_{s},N_{h},N_{v}$ represent the number of satellites, HAPs and UAVs, respectively. $Y_{c,n,m}$ represents the number of links that can be established between nodes n and m at the state c.

To avoid wasting link resources, the MLSCN prohibits data from being transferred back to the lower space nodes from higher space nodes, which is shown as follows:

(2) $$X_{c,n,m}^{y,z}=0\;\;\forall y,\;z,\;(n=s,m=h),\;(n=h,m=v)$$

where $X_{c,n,m}^{y,z}$ represents the flow from node *y* to *z*, and traversing from node *n* to node *m* at state *c*. The changes of the node load in the network must satisfy the flow balance as shown in Equation (3):

(3) $$B_{c,n}^{y,z}-B_{c-1,n}^{y,z}=\sum_{m=1}^{N_{s}+N_{h}}X_{c,m,n}^{y,z}-\sum_{m=1}^{N_{s}+N_{h}}X_{c,n,m}^{y,z}$$

where $B_{c,n}^{y,z}$ represents the number of packets from node y to z which is temporarily stored in node *n* at state *c*.

Assuming that the bandwidth of each contact is constant and equal, then the contact time of each state can also be called the contact capacity [17]. Since the contact capacity of each state is limited, the amount of each flow delivered at state *c* should be less than the contact capacity *CT_c_*, which is shown in Equation (4):

(4) $$X_{c,n,m}^{y,z}\leq CT_{c}$$

### 2.3. Problem Description

In a traditional terrestrial fixed network, the links between node pairs are hardly changed, and the classical shortest path algorithm can efficiently perform data transfer tasks. However, in a time-varying network where the resources of nodes are limited, not only does the topology of the network change dynamically, but also the constraints on the resources make it impossible for the nodes to utilize all the potential contacts. If the shortest path algorithm is directly applied at this time, it is likely that the effective data transfer path cannot be obtained, and the network performance is severely degraded.

**Figure 3:**
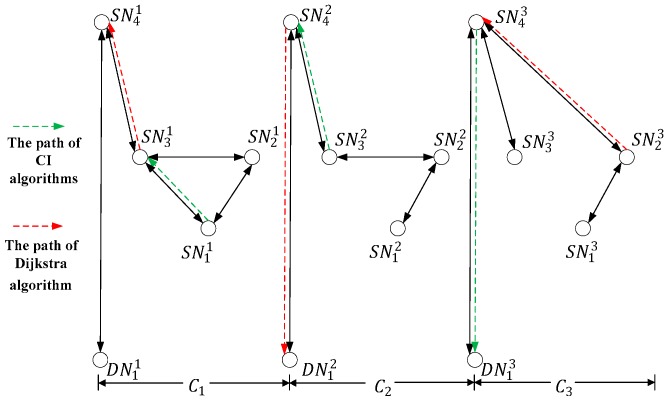
Analysis of CPD Problems in an example space network.

Considering that the large amount of spatial nodes in our model, the example network collectively refers to all spatial nodes as SN in Figure 3, and the destination node is called as DN. With the concern of the time-varying nature of the network topology and the difference in each state duration, the example network is divided into three states $C_{1}$, $C_{2}$ and $C_{3}$. $SN_{1}^{1}$ indicates the spatial node $SN_{1}^{1}$ at state $C_{1}$. For simplicity, the unit of state capacity is set as the time delivering a packet. The contact capacity of $C_{1}$, $C_{2}$ and $C_{3}$ are set to 1, 3, and 3, and there are two packets to be delivered from $SN_{1}^{1}$, $SN_{2}^{1}$, and $SN_{3}^{1}$ to the destination node $DN_{1}^{1}$.

The classic Dijkstra algorithm calculates the shortest path based on the current topology. Since $SN_{1}$ is the closest load node from $DN_{1}^{}$ at state $C_{1}$ and $C_{2}$, then contacts $\left[SN_{3}^{1},SN_{4}^{1}\right]$ and $\left[SN_{4}^{2},DN_{1}^{2}\right]$ are established at the first two states in the algorithm. Because of the limitation on contact capacity at state $C_{1}$, the network can deliver only one packet to the destination node at the first two state. At the last state of the network, the node that can be connected with node $DN_{1}^{}$ has no data to transmit, and it can only establish the contact with other load spatial nodes. Finally, only one packet can be transferred to the destination node within three states.

On the contrary, if CPD is performed in a searching manner using the CI algorithms, the contact established at state $C_{1}$ might not be $\left[SN_{3}^{1},SN_{4}^{1}\right]$ but $\left[SN_{1}^{1},SN_{2}^{1}\right]$. Moreover, $SN_{3}^{2}$ can fully utilize the contact capacity of state $C_{2}$, and transmit three packets to node $SN_{4}^{2}$, so as to enable node $SN_{4}^{3}$ to transmit three packets to the destination node $DN_{3}^{1}$ at state $C_{3}$.

The above example explains why the traditional shortest path algorithm is not suitable for the resource-constrained spatial networks. However, it should be noted that there is more than one type of spatial node in our MLSCN model, and the characteristics of the links between nodes are not the same. Therefore, the inter-layer CPs and intra layer CPs of the MLSCN are designed separately in the following.

## 3. MPSO for Inter-Layer Contact Plan

Considering the complexity of the MLSCN topology and the NP-hardness of the CPD problem itself, we combine the characteristics of the CPD in MLSCN model and the idea of Particle Swarm Optimization (PSO) to propose MPSO and so as to improve the transmission efficiency in inter-layer contacts. Before describing MPSO in detail, several terms used in MPSO are explained here:

*Particle*: Particles are individuals who perform the optimization process in MPSO. They adjust their speed and position through historical information to gradually approach the optimal solution in an iterative way. A particle is represented by a string of binary bits.

*Group*: A group consists of several particles, and the number of particles is called as group size.

*Learning factor*: The learning factor adjusts the particle trajectory through historical information. The larger the parameter, the greater the influence of the historical information.

*Inertia weight*: The inertia weight represents the influence degree of the current speed on the trajectory. The larger the parameter, the stronger the global search ability of particles.

**Figure 4:**
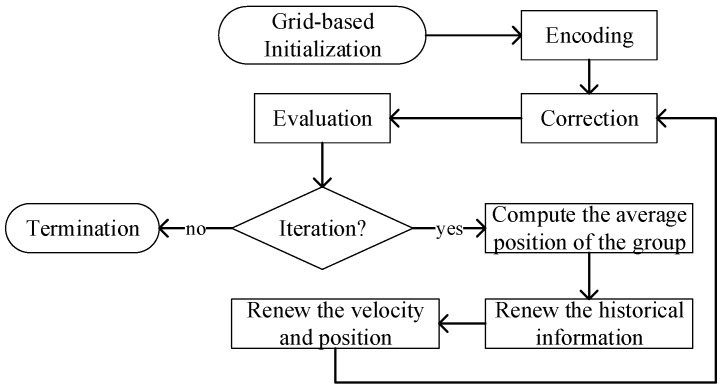
The flow chart of MPSO.

The overall process of MPSO is shown in Figure 4. The algorithm first initializes the group by grid-based initialization, which is aimed to increase the diversity of the group by minimizing the relevancy between particles. Then, the particles are coded to bit strings according to the initialized group. However, the generated CPs may not meet the requirement of system, so the next step is to repair each particle. The repaired particle can be regarded as a feasible CP, then MPSO evaluates each particle by an evaluation function so as to distinguish the merits of each particle. 

To this point, MPSO completes current iteration and determines whether to continue iterating. If MPSO chooses to continue iterating, then it enters the main optimization step of the algorithm. Through the worst and best position of the particle, after this, MPSO guides the direction of motion of the particles through the worst and best position saved in the iterative process, and gradually approaches the optimal solution of the system. In fact, updating the code string only by the direction guidance may break the delivery requirement of system. Thus, particles that have just finished the position update need to be repaired again. The specific implementation of each part of MPSO is described below.

Traditionally, heuristic algorithms initialize the group by random binary strings. Although this method can obtain an initial group with a low computational overhead, randomly generated strings may cause the particles to be unevenly distributed in the solution domain, and quickly fall into the local optimization trap in the following iteration process. In light of this, the grid-based in MPSO is introduced to disperse the particles in the initial group as much as possible. 

**Figure 5:**
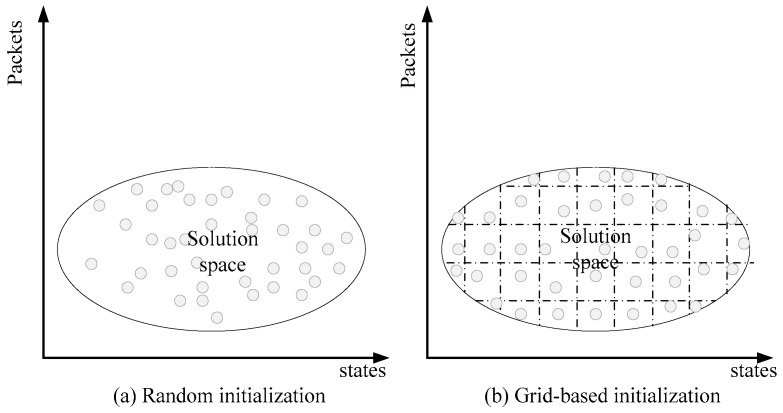
Random initialization and Grid-based initialization with two dimensions in MPSO.

Since only the UAVs are source data nodes in the MLSCN model, the packets mush be delivered to the HAPs before transmitted to satellites through SPLs. Thus, the grid-initialization here is the initialization of the PVLs. In addition, it should be noted that Figure 5 shows the solution domain of the same PVL, rather than the solution domain of a complete CP in the MLSCN. In other words, a CP has multiple solution domains in Figure 5, and the points in the figure indicate the PVL in a CP, which is characterized by the average establishing time and the amount of data. Figure 5a shows the establishment of a PVL in the initial group generated by the random initialization, in which many of these PVLs have similar average establishing time and data amount. To maximize the link diversity in each CP, grid-based initialization in Figure 5b distributes PVLs uniformly in two dimensions. The MPSO initializes the group by the following steps:

*Step 1*: Based on the contact topology of MLSCN, a certain number of CPs that can complete the data transmission on HAPs are randomly generated, and the average delivery time of these CPs is calculated at the same time.

*Step 2*: According to the average delivery time calculated in step 1 and the contact topology of MLSCN, MPSO counts all PVLs that may be established during the delivery period, and calculates the average establishing time and the transfer amounts of the contact.

*Step 3*: MPSO calculates the relevancy between each pair of CPs according to the average values calculated in step 2.

*Step 4*: Based on the optimization model of relevancy minimization, the classical Genetic Algorithm (GA) is used to iteratively correct the transfer amounts and average establishing time of each PVL in CPs until the iteration termination condition is reached.

Due to the needs of the average establishing time and transfer amounts of PVLs, the first step of gird-based initialization is to generate a large number of CPs randomly, and so as to determine the delivery period on HAPs. After this, the average establishing time and the average transfer amounts of each link are calculated according to Equations (5) and (6):

(5) $$X_{n,m}^{ave}=\frac{\sum_{pl=1}^{N_{pl}}\sum_{t=1}^{T^{ave}}X_{t,n,m}^{pl}}{N_{pl}}\;\;n=v,m=h$$

where $X_{n,m}^{ave}$ is the average amount of data flowed in the contact [n,m], and $N_{pl}$ is the group size. pl indicates the identifier number of a CP, which is smaller than $N_{pl}$. $X_{t,n,m}^{pl}$ represents the amount of data delivered by contact [n,m] in CP pl. $T^{ave}$ is the average time it takes to transfer all the data on UAVs, which is calculated by Equation (6):

(6) $$T_{n,m}^{ave}=\frac{\sum_{pl=1}^{N_{pl}}T_{n,m}^{pl}}{N_{pl}}\;\;n=v,m=h$$

where $T_{n,m}^{ave}$ indicates the average establishing time of contact [n,m] in all CPs in the group. $T_{n,m}^{pl}$ is the average establishing time of contact [*n,m*] in CP *pl*, which is calculated by Equation (7):

(7) $$T_{n,m}^{pl}=\frac{\sum_{t=1}^{T^{ave}}T_{t,n,m}^{pl}}{\sum_{t=1}^{T^{ave}}CT_{t,n,m}^{pl}}\;\;\;\forall pl,n=v,m=h$$

where $T_{t,n,m}^{pl}$ indicates the current establishing time of contact [*n,m*] in CP *pl*, and $\sum_{t=1}^{T^{ave}}CT_{t,n,m}^{pl}$ expresses the cumulative contact capacity of contact [*n,m*] in CP *pl*.

The system evaluates the relevancy of the current initial group after calculating the characteristics of the PVL. The formula is as follows:

(8) $$r(pl_{i},pl_{j})=\frac{Cov(X^{pl_{i}},X^{pl_{j}})}{\sqrt{Var[X^{pl_{i}}]\cdot Var[X^{pl_{j}}]}}+\frac{Cov(T^{pl_{i}},T^{pl_{j}})}{\sqrt{Var[T^{pl_{i}}]\cdot Var[T^{pl_{j}}]}}$$

where *pl_i_* and *pl_j_* indicate the *i*th and jth CP in the initial group. $Cov(X^{pl_{i}},X^{pl_{j}})$ and $Cov(T^{pl_{i}},T^{pl_{j}})$ are the covariance of CP *pl_i_* and CP *pl_j_* in terms of the data amount and the average establishing time of, which can be calculated by the following Equations (9) and (10). $Var[X_{n,m}^{pl_{i}}]$ and $Var[T_{n,m}^{pl_{j}}]$ are the variance of $X_{n,m}^{pl_{i}}$ and $T_{n,m}^{pl_{j}}$, which can be calculated by Equations (11) and (12):

(9) $$Cov(X^{pl_{i}},X^{pl_{j}})=\sum_{n=1}^{N_{n}}\sum_{m=1}^{N_{m}}(X_{n,m}^{pl_{i}}-X_{n,m}^{ave})\cdot \sum_{n=1}^{N_{n}}\sum_{m=1}^{N_{m}}\left(X_{n,m}^{pl_{j}}-X_{n,m}^{ave}\right)\;\;\;n=v,m=h,\forall i\neq j$$

(10) $$Cov(X^{pl_{i}},X^{pl_{j}})=\sum_{n=1}^{N_{n}}\sum_{m=1}^{N_{m}}(T_{n,m}^{pl_{i}}-T_{n,m}^{ave})\cdot \sum_{n=1}^{N_{n}}\sum_{m=1}^{N_{m}}\left(T_{n,m}^{pl_{j}}-T_{n,m}^{ave}\right)\;\;\;n=v,m=h,\forall i\neq j$$

where $X_{n,m}^{pl_{i}}$ is the amount of delivered data on contact [*n,m*] in CP *pl_i_*, and $T_{n,m}^{pl_{i}}$ is the average establishing building time of contact [*n,m*] in CP *pl_i_*:

(11) $$Var(X^{pl})=\frac{\sum_{n=1}^{N_{n}}\sum_{m=1}^{N_{m}}{\left(X_{n,m}^{pl}-X_{n,m}^{ave}\right)}^{2}}{N_{T^{ave}}^{l}}\;\;\;\forall pl,n=v,m=p$$

(12) $$Var(T^{pl})=\frac{\sum_{n=1}^{N_{n}}\sum_{m=1}^{N_{m}}{\left(T_{n,m}^{pl}-T_{n,m}^{ave}\right)}^{2}}{N_{T^{ave}}^{l_{n,m}}}\;\;\;\forall pl,n=v,m=p$$

where $N_{T^{ave}}^{l_{n,m}}$ is the number of contacts that MLCN can establish by the time *T^ave^*.

To maximize the differences among the particles of the group, the objective function in grid-based initialization is set as Equation (13):

(13) $$\min\sum_{i}\sum_{j}r(pl_{i},pl_{j})\;\;\forall i\neq j$$

According to the objective function, the classical Genetic Algorithm (GA) is used to search the optimal solution of initial group. Table 1 shows the simulation result, the comparison between traditional way and grid-based initialization in relevancy.

As can be seen in Table 1, whether for transfer amounts or average establishing time, the initialized group is much smaller than that of random initialization.

The initialized MPSO encodes each CP in the initial group into a binary string leading the algorithm more manageable. Each bit of the string represents an establishment status of a particular contact. In the example contact topology of Figure 2, considering the cross-layer contacts $\left[v_{2}^{1},p_{1}^{1}\right]$ and $\left[v_{3}^{1},p_{2}^{1}\right]$ at state $c_{1}$, the binary string reserves two code bits for state $c_{1}$. If the first bit in the binary string equals to 1, it means that contact $\left[v_{2}^{1},p_{1}^{1}\right]$ is established in the CP. Otherwise, the contact is excluded from the CP.

Since the CPD of SPLs is not covered in the grid-based initialization of MPSO, and randomly generated bits are likely to break the constraints of the model, the bits of SPLs must be repaired soon. The repaired string is a complete inter-layer CP, and then combined with the intra-layer CP introduced in the next section, it constitutes a complete feasible CP in the MLSCN. 

In order to demonstrate the advantages and disadvantages of CPs, the following evaluation function is used to calculate the fitness of CPs:

(14) $$F=\frac{1}{\sum_{c}\sum_{n}\sum_{m}CT_{c}\cdot X_{c,n,m}}\cdot \phi \left(T_{s}\right)\;\;\;\forall c,n,m=g$$

where $T_{s}$ is the lifetime of a packet, $\phi \left(T_{s}\right)$ is the penalty function and shown as follows:

(15) $$\phi (T_{s})=1-(\frac{N_{o}^{p}\cdot (T_{o}^{ave}-T_{s})}{N_{all}^{p}\cdot T_{all}^{ave}})$$

where $N_{o}^{p}$ is the number of packets whose delivery time exceeds the lifetime. $T_{o}^{ave}$ is the average delivery time of the packet which exceeds the lifetime, and $T_{all}^{ave}$ is the average time for all packets to reach the destination node. $N_{all}^{l}$ is the total number of the packets waiting delivery on all UAVs. *α* is the penalty factor.

After the evaluation of each particle, MPSO determines whether iterating or not. A static termination condition is adopted in the algorithm, that is if a certain number of iterations is reached, the algorithm will terminate, otherwise it enters the optimization process of MPSO again.

By comparing the current position with the optimal position, the search direction of particles in the traditional PSO [38] can be correctly guided. However, the single form of the PSO may cause particles to quickly fall into local optimization traps, and it is difficult to obtain a good solution. In light of this, MPSO expands the optimization direction by a quaternary optimization strategy. In other words, the search direction of the particles in MPSO can be corrected by the optimal position as well as the worst position. Since the optimization requires historical information, the algorithm then judges whether to update the historical information after the evaluation is completed. The algorithm updates the historical extremum by comparing the current fitness with the worst and the optimal historical fitness, and also reserves the corresponding position of the fitness.

The constraints on the number of transponders will cause a large proportion of zeros in a binary string. If the search direction of the particles is reversely adjusted by the historical worst position, most of the bits in the string will change to 1, which means that the constraints will be broken by the renewed string. And finally, MPSO will repair the strings in a large scale, which will greatly affect the performance of the optimization. To this end, the algorithm calculates the average position of the current group by Equation (16) and uses the calculated position to pick out the bits in the worst string which can correctly guide the search direction. Specifically, at a certain position of a string, if the bit of the worst position is different from the mean value, the bit will have a guiding effect on the direction of the current particle, and vice versa. The average value of the *j*th bit in the current group is calculated in Equation (16):

(16) $$b_{ave,j}^{}=\{\begin{array}{ll} 1 & \sum_{pl=1}^{N_{pl}}b_{pl,j}/N_{pl}>0.5\\ 0 & \sum_{pl=1}^{N_{pl}}b_{pl,j}/N_{pl}<=0.5 \end{array}$$

where $N_{pl}$ is the group size and $b_{pl,i}$ is the value of the *j*th bit of CP *pl*.

After traversing all the bits in the string according to Equation (16), the average position of the group is determined. Then the MPSO uses the quaternary optimization to update the search direction and position:

(17) $$v_{pl,i}(c+1)=w_{pl}(c)\cdot v_{pl,i}(c)+a_{1}\cdot r_{1}\cdot [x_{pl,i}^{o}(c)-x_{pl,i}(c)]+a_{2}\cdot r_{2}\cdot [x_{g,i}^{o}(c)-x_{pl,i}(c)]$$

(18) $$v_{pl,i}(c+1)=w_{pl}(c)\cdot v_{pl,i}(c)+a_{1}\cdot r_{1}\cdot [x_{pl,i}(c)-x_{pl,i}^{w}(c)]+a_{2}\cdot r_{2}\cdot [x_{g,i}^{o}(c)-x_{pl,i}(c)]$$

(19) $$v_{pl,i}(c+1)=w_{pl}(c)\cdot v_{pl,i}(c)+a_{1}\cdot r_{1}\cdot [x_{pl,i}^{b}(c)-x_{pl,i}(c)]+a_{2}\cdot r_{2}\cdot [x_{pl,i}(c)-x_{g,i}^{w}(c)]$$

(20) $$v_{pl,i}(c+1)=w_{pl}(c)\cdot v_{pl,i}(c)+a_{1}\cdot r_{1}\cdot [x_{pl,i}(c)-x_{pl,i}^{w}(c)]+a_{2}\cdot r_{2}\cdot [x_{pl,i}(c)-x_{g,i}^{w}(c)]$$

where $w_{pl}(c)$ is inertia weight of particle pl at state c, $v_{pl,i}(c+1)$ is the speed of particle pl at the *j*th bit in state *c*, which is decided by Equation (20). $a_{1}$ and $a_{2}$ are the learning factors, $r_{1}$ and $r_{2}$ are the random numbers in the range of [0,1]. Under the state c, $x_{pl,i}^{b}(c)$ and $x_{pl,i}^{w}(c)$ are the historical optimal and worst positions at *i*th found by particle pl, $x_{g,i}^{b}(c)$ and $x_{g,i}^{w}(c)$ are the historical optimal positions and the worst positions found by the group, respectively.

A random integer in the range of [1,4] is generated to determine the velocity (i.e., search direction). Random values 1, 2, 3, and 4 correspond to Equations (17)–(20), respectively. It should be noted that if the worst position is involved in the velocity update, the algorithm must determine whether the bit in the worst case is equal to the mean value. If the two bits are different, the velocity of the particle corresponding to the bit is updated, and vice versa.

(21) $$w_{i}(c+1)=\{\begin{array}{ll} w_{\min}-\frac{\left(w_{\max}-w_{\min})\times (f_{pl}(c)-f_{\min}(c)\right)}{f_{avg}(c)-f_{\min}(c)} & ,\;f_{pl}(c)>f_{avg}(c)\\ w_{\max} & ,\;f_{pl}(c)>f_{avg}(c) \end{array}$$

where $w_{\max}$ and $w_{\min}$ are the maximum and minimum inertia weights of the input, $f_{avg}(c)$ and $f_{\min}(c)$ are the average fitness and the minimum fitness of the group, and $f_{pl}(c)$ is the fitness of the CP pl at state c.

After the update of velocity, the particle adjusts its position based on the current position and the updated velocity, which is shown in Equation (22):

(22) $$x_{i,j}(c+1)=x_{i,j}(c)+v_{i,j}(c+1)$$

where $x_{i,j}(c+1)$ is the position of *j*th bit within particle pl at the state c+1.

Considering that each bit in the string is ultimately represented by a binary value, but the issue is not considered in the position update. Thus, the particles that have just finished their position update need to approximate the value of each bit by Equation (23):

(23) $$x_{i,j}(c+1)=\{\begin{array}{c} 0\;,\;x_{i,j}(c+1)<0.5\\ 1\;,\;x_{i,j}(c+1)\geq 0.5 \end{array}$$

In addition, the above update of the bits does not take into account the constraint of the model, so MPSO repairs the newly generated string, and converts the string into a feasible inter-layer CP. The above process will continue until the termination condition of the algorithm is met, and the final CP of MLSCN is the historical optimal position.

## 4. Intra-Layer Contact Plan Design

In the envisioned MLSCN, the main concern of intra-layer links is to deliver data to the intra-layer nodes which can connect the ground nodes. However, different network layers play different role in the overall transmission process, and the connection characteristics between nodes are varied with different layers. Therefore, the CPs in the intra-layer link cannot be designed as a whole, but it should be designed layer by layer according to the characteristics of each layer.

### 4.1. Intra-Layer Contact Plan for UAV Layer

In the UAV layer, different locations of the nodes result in different opportunities to connect with HAP nodes. In other words, different UAV nodes own different number of coding bits in the MPSO. For the nodes with more coding bits, it is easier to establish contacts during the iteration process. If the data is not delivered in the UAV layer in this case, the load gap between UAV nodes will gradually expands during the iteration, eventually causing the congestion and affecting system performance. In light of this, a load balancing strategy is introduced in the intra-layer CPD of UAV layer to alleviate the possible congestion, which is shown in Equation (24):

(24) $$\min\sum_{A}\sum_{n}\left(B_{n}^{A}-\frac{\sum_{n}B_{n}^{A},}{N^{A}}\right)$$

where A is the identifier of UAVs cluster, $B_{n}^{A}$ is the load size of node n in cluster A, and $N^{A}$ is the number of UAVs in cluster A.

### 4.2. Intra-Layer Contact Plan for HAP Layer

In the mesh network constructed by HAPs, the contact can only be established continuously between neighboring nodes. To improve the transfer efficiency within HAP layer as much as possible, a modified Dijkstra algorithm is utilized to minimize the number of hops in data transfer. Due to the limited number of transponders, the sequence of the contacts established within the layer may affect transfer efficiency. For example, if the lifetime of the data is short, and if the system prioritizes the establishment of contacts far from the destination node, the closer contacts cannot be established because of the occupation of the transponder, which may make the data unable to reach the destination node on time. Therefore, in the modified Dijkstra algorithm, the contacts are established in the order of hop count from small to large. In other words, the algorithm preferentially delivers the data that close to the destination node.

In addition, in the case of a limited number of transponders, if data is delivered by its own unique shortest path, this may break the constraints and further increase the congestion within the layer. In fact, since the model evaluates the distance from the destination node based on the hop count, each node may have more than one shortest path. Under this consideration, multiple shortest paths are reserved in the modified Dijkstra algorithm. When the data transmission is congested in HAP layer, the algorithm will traverse the next hop nodes of all the shortest paths of the congested node, select an available node and then deliver the data. If all the next nodes are occupied, the data will not be delivered at current state.

### 4.3. Intra-Layer Contact Plan for Satellite Layer

Taking into account the time-varying characteristics of the topology in the satellite network, a classic algorithm named as DVTR [39] is employed to schedule the contacts within the satellite layer. Like the HAP layer, the algorithm also reserves multiple shortest paths. Once the preferred next node is occupied, the available next node of another shortest path is selected to establish the contact. Besides, in order to improve the arrival rate of data, the contact which is closer to the destination node will be given higher priority to be established.

## 5. Simulation and Analysis

### 5.1. Simulation Settings

To verify the validity of our proposed CPD schemes, MATLAB and STK are used for our co-simulation. In the envisioned MLSCN, it consists of satellites, HAPs, UAVs, and ground stations. Specifically, the satellite layer uses the Iridium constellation, six polar orbits with 11 satellites in each orbit. Each satellite can connect with two adjacent satellites in the same orbit and two satellites in the adjacent orbits. The intra-track link can be established continuously, and the inter-track link will be closed above two polar regions [40].

The HAP layer is a mesh network of 5 × 5 nodes in the range of [29.38° N,40.86° N], [85.55° E,113.35° E], and its height is 20 km [37]. A total of three data transfer tasks are performed during simulation, and thus there are three UAV clusters in the network, and each of cluster has 6, 7, and 7 UAV nodes. The flight range of each cluster is a circle with a radius of 200 km, and the centers of the three clusters are (32.32° N, 112.371° E), (40.26° N, 112.55° E) and (38.11° N, 81.55° E), respectively. Three ground stations are separately located at Xi’an (34.45° N, 109.50° E), Mi’yun (40.45° N, 116.86° E), and He’tian (37.11° N, 79.92° E). For simplicity, we set the data transfer rate to 1 Mb/s and the packet size to 1 Mbit [41], which means that the transmission of a packet will occupy one second of a contact. The default lifetime of a packet is set to 4200 s. In the MPSO, the group size is set to 100. The inertia weight, the minimum inertia weight, the maximum inertia weight, the local learning factor, and the global learning factor are set to 0.6, 0.5, 0.8, 2, and 2, respectively. Other parameters of the related satellite network are shown in Table 2.

**Figure 6:**
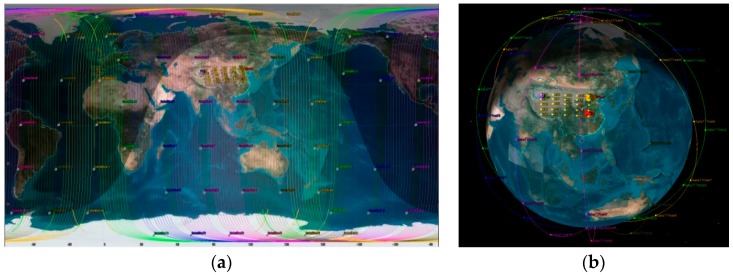
Network architectures in STK. (**a**) 2D network model; (**b**) 3D network model.

The 3D and 2D views of the system network in STK are shown in Figure 6.

### 5.2. Performance Analysis

In this work, we mainly compare the CPD performance of different heuristics in the inter-layer link. Two classic heuristics, viz. Genetic Algorithm (GA) and Particle Swarm Optimization (PSO), are selected as the baseline algorithms. The parameter settings of the baseline algorithms are the same as that of MPSO in this paper, and the iteration times and group size are 500 and 100, respectively. The crossover rate and mutation rate of GA are set to 0.6 and 0.05, and the GA adopts the elitism strategy of the survival of the fittest when picking offspring [42]. The inertia weight in the PSO algorithm is set to 0.6, and the local learning factor and global learning factor of the algorithm are the same as those of MPSO in this paper, which are 2 and 2, respectively. Additionally, to compare all algorithms as fairly as possible, the evaluation function of all algorithms is the same as MPSO.

**Figure 7:**
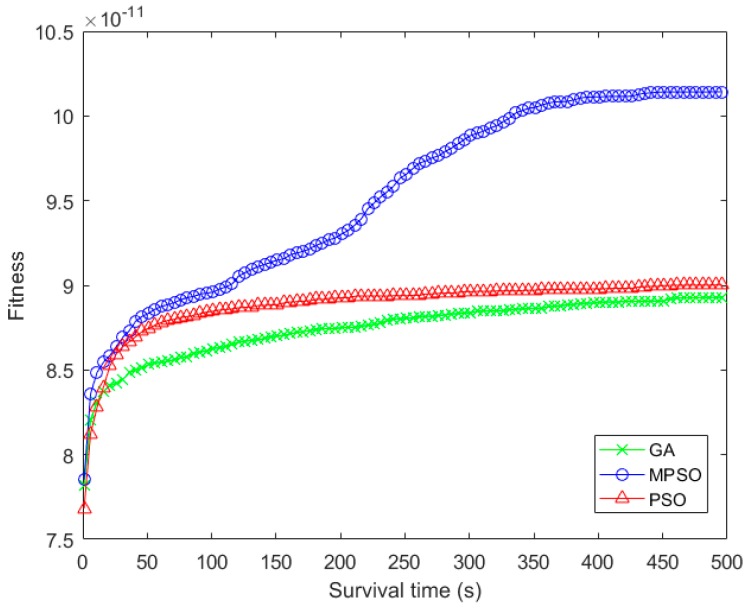
Comparison of fitness with the iteration times.

Since the three algorithms reserve the optimal individual during the iteration process, their fitness gradually increases with the number of iterations in Figure 7. Specifically, the crossover in GA can reorganize the binary strings in a large scale, and increase the diversity of individuals in the group. Therefore, the fitness of GA is lower than PSO in the early stage, while the gap between GA and PSO in fitness gradually narrow with iteration times. Different from the traditional PSO, MPSO relies on the historical optimal and worst positions for adjusting the search direction of particles. This method can fully expand the diversities of individuals and improve the search ability of the algorithm. In addition, the dynamic inertia weight in MPSO enables the algorithm to balance the guidance between the particle historical experience and the self-exploration during the iteration process, and to improve the search ability.

**Figure 8:**
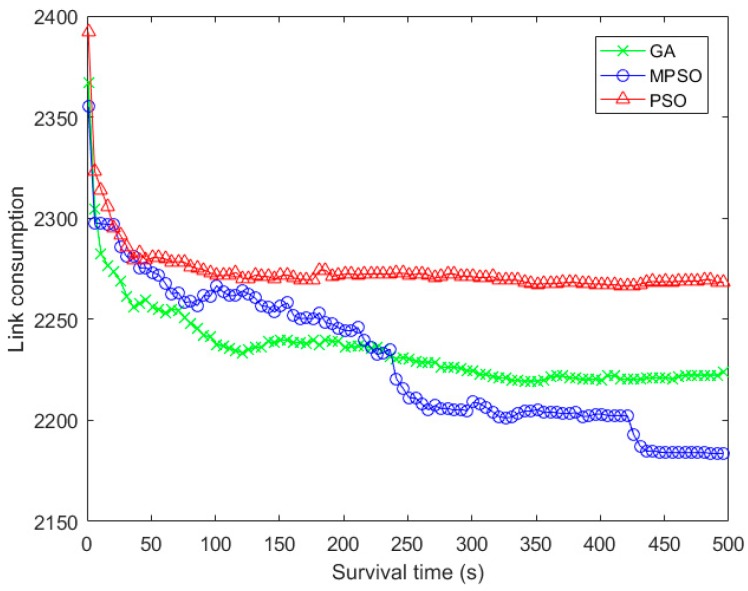
Comparison of link consumption with the iteration times.

In Figure 8, since the PSO does not adopt a targeted optimization strategy for the MLSCN, its link consumption falls into a local optimization trap at the early stage of iteration. On the contrary, in order to expand the diversity among the particles in the initial group, MPSO designs a grid-based initialization and develops a quaternary optimization scheme to guide the optimization efficiently. As a result, the downward trend of link consumption of MPSO is more distinct than that of PSO.

**Figure 9:**
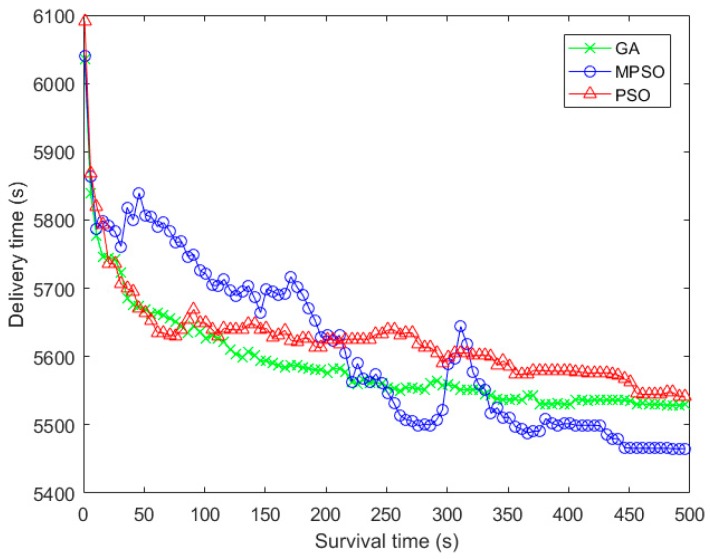
Comparison of delivery time with the iteration times.

In Figure 9, the delivery time refers to the time that it takes to transfer all packets in the network. The delivery time of MPSO shows a trend of fluctuating downward. This can be accounted that the fitness is not only determined by the delivery time, but also closely related to the arrival rate of data. Similarly, it can be seen from the figure that the differences in the delivery time is not as apparent as the difference on fitness. Again, this is because the fitness is not only based on the delivery time, but also the arrival time of each data flow and the arrival rate of data.

**Figure 10:**
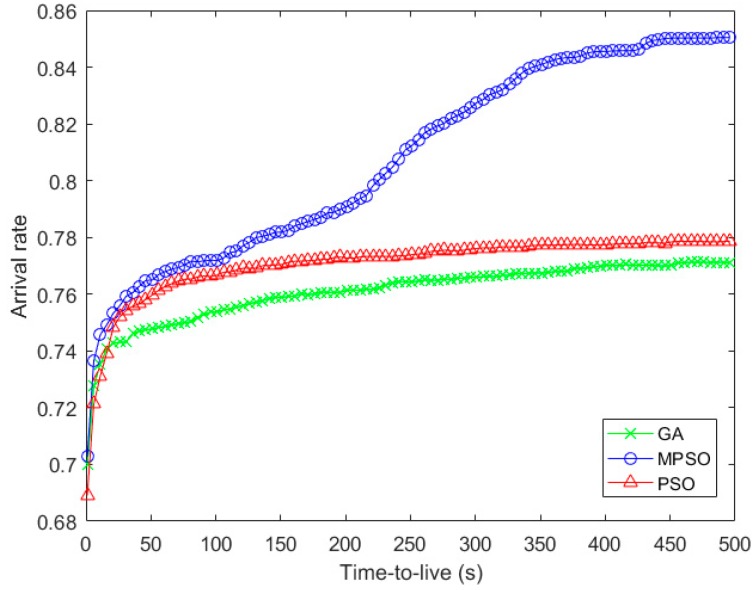
Comparison of arrival rate with the iteration times.

**Figure 11:**
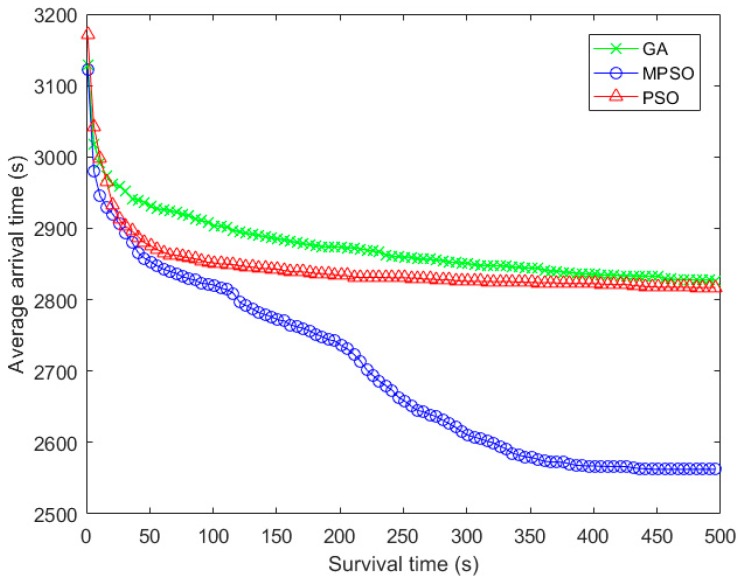
Comparison of average arrival time with the iteration times.

Figure 10 and Figure 11 show the arrival rate and the average delivery time with the iteration times. And the performance trend in Figure 7 and Figure 10 are almost the same, which can be accounted the fact that the arrival rate will greatly affect the fitness when the data cannot be delivered within the lifetime. In terms of average arrival time of packets, the advantage of arrival rate in MPSO makes most of the packets arrive to the destination node earlier, compared with the baseline algorithms.

## 6. Conclusions

In this paper, CP is designed in a MLSCN with the CI method to transfer a large amount of spatial data under limited-resource conditions. Specifically, we have first built a system model to determine the limitations of CPD in the system. Then, this paper divides the contacts in the model into inter-layer contacts and intra-layer contacts, and performs CPD on the two types of contacts respectively according to the order in which the data is delivered in the network. For the inter-layer CP, we have proposed the CI algorithm named MPSO, which introduces the grid-based initialization method to maximize the differences between individuals in the initial group, and at the same time developed a quaternary optimization strategy to further improve the search ability of the algorithm. For the intra-layer contact plan, since each network layer plays a different role in MLSCN, we have designed different intra-layer CPs for different layers. A fair contact plan is designed for the UAV layer, and the HAP and satellite layers are designed with the corresponding CP with the shortest path. Finally, simulation results show that the proposed MPSO can outperform the other two classical CI algorithms.

## Figures and Tables

**Table 1:** The relevancy of grid-based initialization.

	Traditional Way	Grid-Based Initialization
Transfer amounts	0.4004	0.0628
Average building time	0.9592	0.7521

**Table 2:** Orbital parameters in satellite network.

Start time	4 January 2018 04:00
Inclination (degree)	86.4
Height (Km)	780 Km
Orbit Planes	6
Satellites	66
RAAN (degree)	31.6 (Co-directional) 22 (Reverse)
